# Infection with *Mycobacterium tuberculosis* induces the Warburg effect in mouse lungs

**DOI:** 10.1038/srep18176

**Published:** 2015-12-10

**Authors:** Lanbo Shi, Hugh Salamon, Eliseo A. Eugenin, Richard Pine, Andrea Cooper, Maria L. Gennaro

**Affiliations:** 1 Public Health Research Institute, New Jersey Medical School, Rutgers, The State University of New Jersey, Newark, NJ, USA; 2 Knowledge Synthesis Inc. Berkeley, CA, USA; 3 Trudeau Institute, Saranac Lake, NY, USA

## Abstract

To elucidate the little-known bioenergetic pathways of host immune cells in tuberculosis, a granulomatous disease caused by the intracellular pathogen *Mycobacterium tuberculosis*, we characterized infected murine lung tissue by transcriptomic profiling and confocal imaging. Transcriptomic analysis revealed changes of host energy metabolism during the course of infection that are characterized by upregulation of key glycolytic enzymes and transporters for glucose uptake, and downregulation of enzymes participating in the tricarboxylic acid cycle and oxidative phosphorylation. Consistent with elevated glycolysis, we also observed upregulation of a transporter for lactate secretion and a V type H^+^ -ATPase involved in cytosolic pH homeostasis. Transcription profiling results were corroborated by immunofluorescence microscopy showing increased expression of key glycolytic enzymes in macrophages and T cells in granulomatous lesions. Moreover, we found increased mRNA and protein levels in macrophages and T cells of hypoxia inducible factor 1 alpha (HIF-1α), the regulatory subunit of HIF-1, a master transcriptional regulator. Thus, our findings suggest that immune cells predominantly utilize aerobic glycolysis in response to *M. tuberculosis* infection. This bioenergetic shift is similar to the Warburg effect, the metabolic signature of cancer cells. Finding immunometabolic changes during *M. tuberculosis* infection opens the way to new strategies for immunotherapy against tuberculosis.

Immune cell activation is coupled with profound changes in cellular metabolism^1^,^2^. Innate immune cells including neutrophils, dendritic cells, and macrophages switch energy metabolism from oxidative phosphorylation to glycolysis when activated by Toll-like receptor (TLR) ligands or proinflammatory cytokines^3^,^4^. In addition, the metabolic profile of effector T cells involved in inflammation, such as T-helper 17 cells, shows elevated glucose uptake and glycolysis, while oxidative phosphorylation is the dominant energy source in naïve and regulatory T cells^4^,^5^,^6^. Glycolysis not only produces ATP faster than oxidative phosphorylation, albeit less efficiently, but it also provides metabolic intermediates needed for cell growth and proliferation^7^. A shift to glycolysis thus supports a rapid and vigorous inflammatory response to bacteria, involving the generation of ROS and proinflammatory cytokines^4^. Dependence on glycolysis for energy in activated, inflammatory immune cells is akin to the Warburg effect, a metabolic phenomenon best studied in cancer cells^7^. The Warburg effect describes the predominant cellular utilization of aerobic glycolysis with formation of lactate instead of oxidative phosphorylation in mitochondria for the generation of ATP and recycling of NADH to NAD^+^^8^. Numerous studies have indicated that the Warburg effect is mediated by the master transcription factor hypoxia-inducible factor-1 (HIF-1)^9^. In addition to regulating energy metabolism and cellular adaptation under hypoxia, HIF-1 also plays a regulatory role in inflammation under normoxia. Indeed, HIF-1α, the regulatory subunit of HIF-1, is induced by proinflammatory cytokines, growth factors, bacterial products, and viral infection^10^,^11^,^12^,^13^. The relationship between HIF-1, cellular metabolism, and the immune response to infectious agents remains poorly understood.

The host immune response determines the outcome of tuberculosis, a chronic infection caused by the intracellular pathogen *Mycobacterium tuberculosis*. Host-pathogen interactions occur primarily in the lung granuloma, a dynamic and organized aggregate of immune cells comprising a central area of infected macrophages surrounded by other uninfected phagocytic cells and lymphocytes^14^. The immune response in the granuloma contains infection but also causes the tissue damage that results in diminished respiratory function while allowing infection to progress and disseminate^15^. While much effort has been placed into understanding outcome-determining mechanisms of innate immunity and T cell development and activation^16^,^17^,^18^, little is known about the changes of cellular metabolism associated with innate and adaptive immune responses expressed in the tuberculous granuloma.

To unravel immunometabolism as an element of the host response to *M. tuberculosis* infection, we analyzed aspects of host cell central metabolism in infected mouse lung by global transcriptomics, immunohistochemistry and immunofluorescence microscopy. We focused our study on the first thirty days of murine lung infection, using a well-characterized, low-dose aerosol infection protocol^19^. During this time, after initial multiplication, bacteria enter a non-replicative state in response to expression of host adaptive immunity^20^, while granuloma-like lesions evolve^21^. Findings from our study suggest that establishment of chronic infection in the mouse lung is associated with changes indicative of increased glucose uptake and glycolysis, lactate formation and export, and decreased oxidative phosphorylation. This metabolic shift, analogous to the Warburg effect, is concurrent with increased levels of HIF-1α, key glycolytic enzymes and metabolic markers in macrophages and T cells at the granulomatous lesion. This novel understanding of immunometabolism in tuberculosis may open new avenues of investigation into host-directed adjunct anti-tuberculosis therapy.

## Results

In a standard mouse model of low-dose, respiratory *M. tuberculosis* infection, initial bacterial multiplication in lung (acute phase of infection) induces expression of adaptive immunity (day ~14) with consequent cessation of bacterial growth (day ~21). With the expression of adaptive immunity, granuloma-like lesions fully develop and chronic infection is established by day 30^21^. To determine the dynamics of gene expression during the transition from acute to chronic phase of the infection, mouse lung samples were collected at multiple times (0, 12, 18, and 30 days) post-infection, RNA extracted, and host transcripts enumerated by RNA-seq. Measuring the expression of *Ifng*, a marker for adaptive immunity (Supplemental Fig. S1), verified the previously described dynamics of host-pathogen interaction during lung infection^20^. Moreover, the observation that the change of *Ifng* mRNA level assayed by RT-PCR before and after rRNA depletion was similar to that determined by RNA-seq (Supplemental Fig. S1), validated the methodology of RNA-seq. In the present work, we first present RNA-seq data from the first thirty days of *M. tuberculosis* infection for mouse genes involved in central metabolic pathways. Only transcripts exhibiting significant changes (p ≤ 0.05) between day 30 and one or more preceding time points are shown in the sections below. Except as noted, changes in gene expression among these are given for mRNA levels at 30 days post-infection compared to levels from uninfected controls. The gene expression data and statistical analysis for all the genes encoding glucose transporters, glycolytic enzymes, enzymes in the pentose phosphate pathway (PPP), monocarboxylic transporters and subunits of vacuolar H^+^ ATPase (V-ATPase) are shown in Supplemental Table S1.

### Enhanced glucose uptake, glycolytic pathway and the PPP in response to *M. tuberculosis* infection

**Figure 1:**
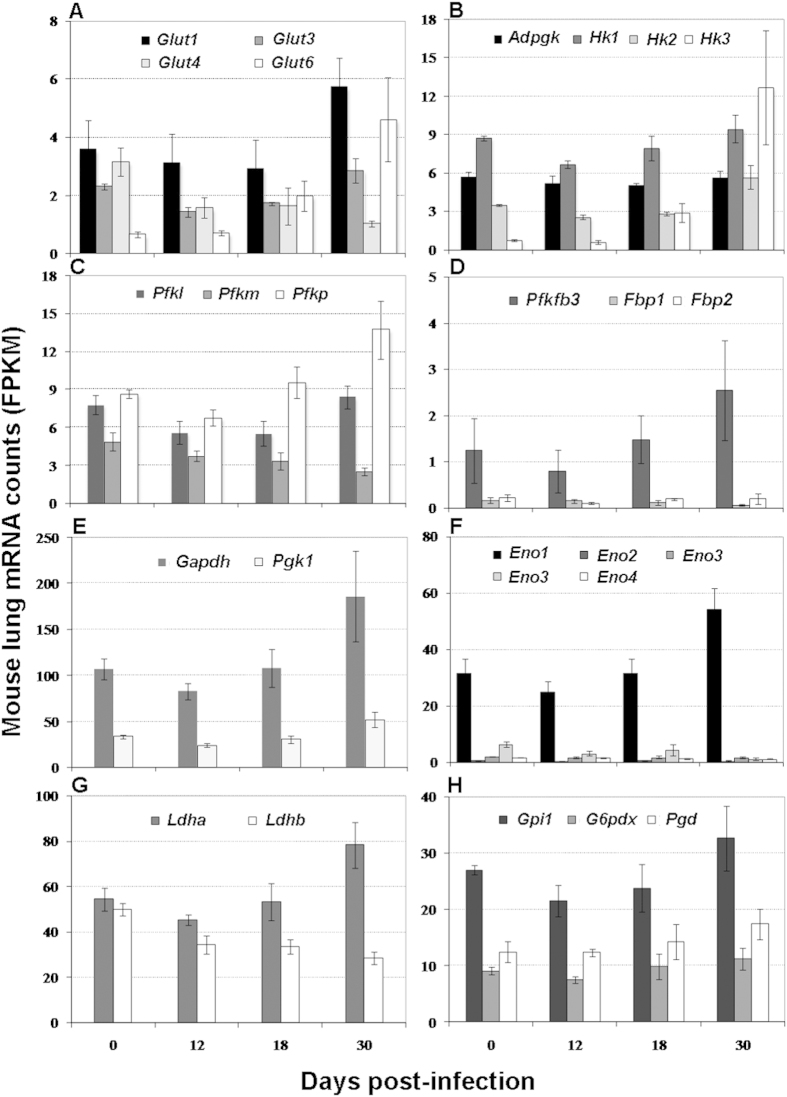
Change of transcripts encoding facilitative glucose transporters, glycolytic enzymes and enzymes in the pentose phosphate pathway in infected mouse lungs. Shown are average ± SDs of the normalized mRNA counts (FPKM, Fragments Per Kilobase of exon per Million fragments mapped) from 3 mice at each time point. Each panel represents expression of select genes encoding facilitative glucose transporters (**A**), glycolytic enzymes (**B–G**), or enzymes of the pentose phosphate pathway (H). Two splice variants are shown for *Eno3.* Changes described in the text are at p ≤ 0.05 (student’s t-test) at corresponding time points.

Upregulation of facilitative glucose transporters. The first limiting step in glucose metabolism is the rate at which glucose is captured and transported into the cell. We examined expression levels of seven genes involved in glucose transport (*Glut1-7*)^22^,^23^,^24^ (Supplemental Table S1). *Glut2* and *Glut4* decreased up to ~3-fold (*Glut5* and *Glut7* were either very low or undetectable). In contrast, *Glut1* and *Glut3* transcripts increased ~2-fold (D30 vs D12), and strong induction (almost 7-fold) was detected for one mRNA splice variant of *Glut6* (the other variant showed very low expression levels) (Fig. 1A). Since *Glut6* transcripts are only exclusively detected in brain and lymphoid tissues^25^, the observed strong induction suggests that GLUT6 is probably a major glucose transporter accompanying the activation and differentiation of immune cells that infiltrate mouse lungs during *M. tuberculosis* infection.

Upregulation of hexokinase 2 and 3. To maintain glucose uptake by the facilitated diffusion mediated by GLUT transporters, a glucose gradient is required across the plasma membrane. This gradient is created by the key regulatory step of glucose metabolism-glucose phosphorylation by hexokinases (HK1–3), glucokinase (GCK), and the ADP-dependent kinase (ADPGK)^26^,^27^. We detected transcripts for all 5 glucose kinases in mouse lungs (Supplemental Table S1). Transcripts for *Hk1* and *Gck* showed little change during infection and *Hk2* transcripts showed >1.5-fold induction (Fig. 1B and Supplemental Table S1). The most important change was observed with *Hk3*, which showed a > 15-fold increase in transcript levels (Fig. 1B). Since HK3 has the highest affinity for glucose and lowest inhibition by its product glucose-6-phosphate (G-6-P) among the three hexokinases^26^, its strong upregulation suggests that HK3 is probably a key glucose phosphorylation enzyme responsible for the enhanced uptake of glucose and its metabolism in infected mouse lungs. This observation is consistent with the enhanced glucose uptake in THP1 cells upon *M. tuberculosis* infection^28^.

Upregulation of isoforms of phosphofructokinase 1 and 2. We next examined the irreversible rate-limiting step of glycolysis, in which phosphofructokinase 1 (PFK-1) catalyzes conversion of fructose-6-phosphate (F-6-P) to fructose-1,6-bisphosphate (F-1,6-BP)^29^. When we analyzed all three PFK-1-encoding genes (*Pfkm*, *Pfkl*, and *Pfkp*)^30^, we observed increased expression (~1.5-fold) for *Pfkp,* and *Pfkl* transcripts (D30 vs D12), and decreased expression for *Pfkm* (almost 2-fold) (Fig. 1C). PFK-1 can be potentially inhibited by its product and by ATP, thus limiting carbon flux through glycolysis, but expression of the bifunctional PFK-2 family of 6-phosphofructo-2-kinases/fructose-2,6-bisphosphatases (PFKFB1-4) overcomes the inhibition^31^. While all isozymes of PFK-2 family catalyze the formation of fructose-2,6-bisphosphate (F-2,6-BP), which serves as an allosteric activator of PFK-1 and an inhibitor of gluconeogenic F-1,6-biphosphatase (FBP)^32^, each isozyme has a unique kinase:phosphatase ratio that determines the level of F-2,6-BP^31^. When we analyzed expression of *Pfkfb1-4* during lung infection, we found that one splice variant of *Pfkfb3* increased (>3-fold) (D30 vs D12) (Fig. 1D and Supplemental Table S1). This upregulation agrees with the observation that macrophage activation by LPS and IFN-γ is accompanied by a switch from PFKFB1 to PFKFB3^33^. Since PFKFB3 has the highest kinase:phosphatase ratio of all isozymes in the family^34^, its upregulation should favor accumulation of F-2,6-BP, which in turn would enhance glycolysis by activating PFK-1 and inhibiting gluconeogenic FBP. Indeed, of the two FBPs, *Fbp1* transcript levels decreased (>2.5-fold) (Fig. 1D). Together, upregulation of *Pfkfb3* and of the PFK-1 isozyme genes *Pfkp* and *Pfkl*, along with downregulation of *Fbp1*, should increase carbon flux through glycolysis.

Upregulation of glyceraldehyde 3-phosphate dehydrogenase and phosphoglycerate kinase 1. We then analyzed transcripts for glyceraldehyde 3-phosphate dehydrogenase (GAPDH) and phosphoglycerate kinase 1 (PGK1), which catalyze sequential reactions from G-3-P to the formation of 3-phosphoglycerate (3-PG). Both transcripts were induced (1.5-2.0 fold) during infection (Fig. 1E). A similar scenario is observed in cancer cells^35^,^36^, strongly suggesting that *Gapdh* and *Pgk1* upregulation might contribute to increased flux through glycolysis. Moreover, since GAPDH has non-glycolytic functions such as redox sensing and regulation^37^, its upregulation may also have a role in generating NADPH for oxidative stress response.

Upregulation of enolase 1. Enolase (ENO), another key glycolytic enzyme, catalyzes the conversion of 2-phosphoglycerate (2-PG) to phosphoenolpyruvate (PEP). The three subunits of enolase 1–3 (encoded by *Eno1, 2 and 3*) combine to form various isozymes^38^. Moreover, an enolase-like protein is encoded by *Eno4* in mice^39^. The most expressed *Eno1* transcript was induced (almost 2-fold), while *Eno4* and one *Eno3* splice variant were similarly decreased (1.5- to 2.0-fold) (no changes were seen with the remaining *Eno* transcripts) (Fig. 1F and Supplemental Table S1). Since *Eno1* is upregulated in cancer cells in association with elevated glycolysis and cell proliferation and invasion^40^, its upregulation during *M. tuberculosis* infection of the mouse lung also suggests increased carbon flux through glycolysis in host cells.

Upregulation of lactate dehydrogenase A. To sustain increased glycolysis, pyruvate must be converted to lactate with concomitant recycling of NADH to NAD^+^ for redox homeostasis. Functional lactate dehydrogenases are assembled in different tissues into catalytically active homo- and hetero-tetramers of protein subunits encoded by *Ldha*, *Ldhb* and *Ldhc*, respectively^41^. One highly expressed *Ldha* mRNA splice variant was induced and the *Ldhb* transcript was decreased ~1.5-fold (Fig. 1G) (*Ldhc* expression was not detected). This result suggests that LDHA is the dominant subunit of the lactate dehydrogenase expressed in the infected mouse lung, as also seen with cancer cells and activated immune cells^42^,^43^.

Upregulation of the PPP. We also analyzed the transcription profiles of genes involved in the PPP with the consideration that G-6-P from glucose phosphorylation also serves as substrate for the PPP. We found that genes (*Gpi1* and *G6pdx* (D30 vs D12), and *pgd*)) encoding key enzymes of this pathway were induced by up to 1.5 folds (Fig. 1H and Supplemental Table S1). This observation is consistent with the finding that M1 macrophage is associated with increased carbon flux to glycolysis and the PPP^44^. Since the PPP provides NADPH and riboses for reductive biosynthesis, its upregulation suggests enhanced host cellular anabolic activities in response to *M. tuberculosis* infection.

### Downregulation of pyruvate dehydrogenase complex in response to *M. tuberculosis* infection

When pyruvate is not converted to lactate in the cytosol, it is predominantly oxidized in mitochondria by the pyruvate dehydrogenase complex (PDC) to acetyl CoA. PDC, which is key in glucose homeostasis, consists of E1-pyruvate dehydrogenase α (PDHα) and β (PDHβ), E2-dihydrolipoamide S-acetyltransferase (DLAT), and E3-dihydrolipoamide dehydrogenase (DLD). We found that transcript levels for *Pdhα, Pdhβ, and Dlat* decreased 25–40% during infection (Supplemental Fig. S2), indicative of decreased carbon flux through pyruvate oxidation in the mitochondria.

### Downregulation of tricarboxylic acid cycle and oxidative phosphorylation in response to *M. tuberculosis* infection

To evaluate how decreased pyruvate oxidation relates to the activity of tricarboxylic acid (TCA) cycle and subsequent oxidative phosphorylation in mitochondria, we next analyzed expression profiles of genes encoding enzymes and proteins involved in TCA cycle, electron transport chain (ETC), and ATP synthesis. Transcripts encoding many enzymes in the TCA cycle and components of the ETC and ATP synthase were down-regulated in the infected mouse lung (Supplemental Fig. S3). These results suggest decreased activity of TCA cycle and oxidative phosphorylation, in agreement with diversion of carbon flux toward glycolysis with the formation of lactate in cytosol.

### Upregulation of pathways maintaining cytosolic pH homeostasis in response to *M. tuberculosis* infection

**Figure 2:**
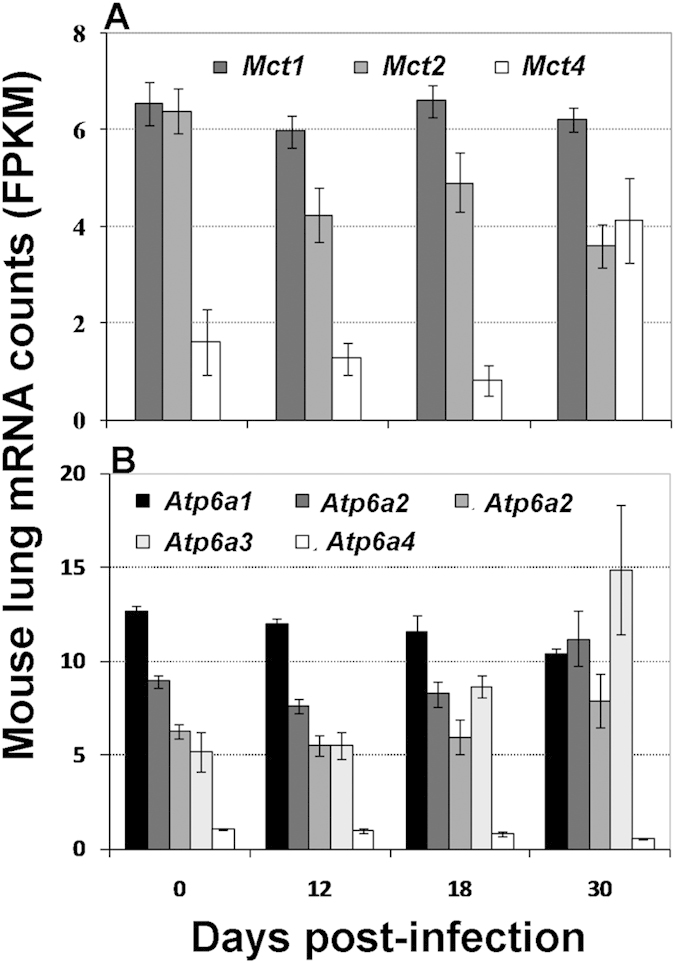
Change of select transcripts mediating cytosolic pH homeostasis in infected mouse lungs. Shown are average ± SDs of the normalized mRNA numbers (FPKM) from 3 mice at each time point. Each panel represents expression of select genes encoding monocarboxylate transporters (MCTs) (**A**) or subunits of V0 subunit a of V-ATPase (**B**). Two splice variants are shown for *ATP6a2.* Changes described in the text are at p ≤ 0.05 (student’s t-test) at corresponding time points.

Without pH homeostatic mechanisms, glycolytic acidosis due to the formation of lactate through enhanced glycolysis would compromise cell viability. To maintain cytosolic pH homeostasis, mammalian cells express a family of monocarboxylate transporters (MCTs) and V-ATPases that transport protons across both intracellular and plasma membranes of some specialized cells. Among the MCTs, MCT1 to MCT4 (encoded by *Slc16a1*, *Slc16a7*, *Slc16a8,* and *Slc16a3*, respectively) play a dominant role in proton-linked transport of lactate, pyruvate and ketone bodies^45^, and have specific tissue distribution and substrate properties^45^,^46^. Among them, only one splice variant of *Mct4* increased in copy number (almost 3-fold), while *Mct2* transcripts decreased (almost 2-fold) (Fig. 2A). All other *Mct* transcripts were either expressed at low levels or remained unchanged (Fig. 2A and Supplemental Table S1). These results suggest that MCT4 is the major lactate transporter, which is consistent with the finding that *Mct4* is upregulated in macrophages upon TLR2 and TLR4 stimulations and required for glycolytic reprogramming and proinflammatory response in macrophages^47^.

V-ATPases are composed of ATP-hydrolyzing (V1) and proton-translocating (V0) domains, and their sub-cellular localization is determined by the four isoforms of V0 subunit a (ATP6V0A1-4). V-ATPases containing the a1 and a2 isoforms localize primarily to intracellular compartments, whereas V-ATPases with a3 and a4 isoforms target V-ATPases to the plasma membrane of specialized cells^48^. When we analyzed expression of the V0 domain subunit a isoforms (ATP6a1-4), we found that transcripts for three isoforms were either decreased (up to 2-fold) (*ATP6a1*, *ATP6a4*), or increased (~1.5-fold) (*ATP6a2*) (D30 vs D12); one highly expressed mRNA splice variant for *ATP6a3* was increased (~ 3-fold) (Fig. 2B and Supplemental Table S1). Since expression of *ATP6a3* isoform is associated with targeting the V-ATPase to the plasma membrane^48^ and increases on the cell membrane during T cell activation (initially identified as TIRC7)^49^, its induction suggests enhanced proton export in mouse lung lymphocytes during *M. tuberculosis* infection.

### Switch of energy metabolism to glycolysis (the Warburg effect) of host cells in response to *M. tuberculosis* infection

**Figure 3:**
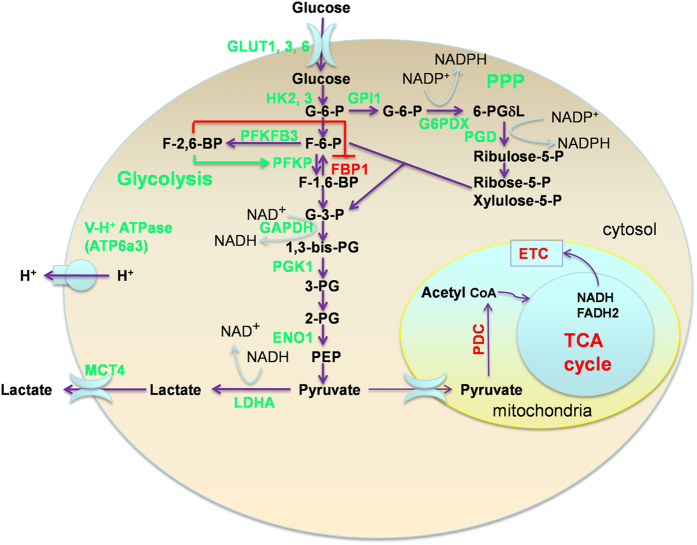
The Warburg effect in *M. tuberculosis*-infected mouse lungs. Schematic presentation of the changes of key enzymes/proteins in central metabolism leading to enhanced carbon flux to glycolysis and subsequent pH regulation in immune cells. Green: upregulated; and red: downregulated. Green arrows: stimulation; and red blocked lines: inhibition. Abbreviations: PPP: pentose phosphate pathway; ETC: electron transport chain; and TCA: tricarboxylic acid cycle. Enzymes or transporters: GLUT1, 3, 6: facilitative glucose transporter member 1, 3, and 6, respectively; HK2-3: hexokinase 2 and 3, respectively; PFKP: phosphofructokinase 1, platelet isozyme; PFKFB3: 6-phosphofructo-2-kinase/fructose-2,6-bisphosphatase 3; FBP1: fructose bisphosphatase 1; GAPDH: glyceraldehyde phosphate dehydrogenase; PGK-1: phosphoglycerate kinase 1; ENO1: enolase 1; LDHA: lactate dehydrogenase A; MCT4: monocarboxylate transporter member 4; ATP6a3: ATPase, H^+^ transporting, V0 protein a3; PDC: pyruvate dehydrogenase complex; GPI1: glucose phosphate isomerase 1; G6PDX: glucose-6-phosphate dehydrogenase X-linked; and PGD: phosphogluconate dehydrogenase. Metabolites: G-6-P: glucose-6-phosphate; F-6-P: fructose-6-phosphate; F-1,6-BP: fructose-1,6-bisphosphate; DHAP: dihydroxyacetone phosphate; F-2,6-BP: fructose-2,6-bisphosphate; G-3-P: glyceraldehyde-3-phosphate; 1,3-bisPG: 1,3-bisphosphoglycerate; 3-PG: 3-phosphoglycerate; 2-PG: 2-phosphoglycerate; PEP: phosphoenolpyruvate; and 6-PGδL: 6-Phosphonoglucono-D-lactone.

As the core of cellular metabolism, central metabolism plays crucial housekeeping functions by providing energy and biosynthetic precursors for various cellular processes, and is usually regulated at posttranslational levels to maintain a relative metabolic steady state^50^. However, the observed statistically significant transcriptional changes in many genes of the central metabolism indicate that pathogen-triggered host immune response is accompanied with a change of host central metabolism at the transcription level. Despite changes in some of these genes are modest, they collectively suggest a metabolic reprogramming in host cells, which is marked with a switch to the predominant use of glycolysis in the cytosol as bioenergetic strategy. We propose that this switch is achieved by the coordinated upregulation of genes encoding isozymes of glucose transporters and glycolytic enzymes (i.e., GLUT6, HK2-3, PFKP, PFKFB3, PGK1, ENO1, and LDHA) and a concurrent downregulation of genes encoding enzymes involved in pyruvate oxidation, TCA cycle and oxidative phosphorylation (Fig. 3). Notably, the induced glycolytic isozymes include those that should accomplish bypass or relief of allosteric inhibition, allowing maximal carbon flux to lactate formation^7^,^51^. Indeed, accumulation of lactate has been observed by ^1^H NMR-based metabolomic profiling in lungs of mice and guinea pigs infected with *M. tuberculosis*^52^,^53^,^54^. Consequently, potential glycolytic acidosis due to enhanced lactate formation would be prevented by induction of selected isoforms of lactate exporters (MCT4) and cell membrane V-ATPase, thus achieving cytosolic pH homeostasis (Fig. 3). The concurrent upregulation of the PPP in host cells, together with the enhanced glycolysis, is in agreement with the metabolic signature of cancer cells^55^. Considering the fact that the transcriptome data are derived from the whole lung, we analyzed the abundance and cellular distribution of tell-tale proteins in *M. tuberculosis*-containing granulomatous lesions by immunofluorescence microscopy to test our gene-expression-generated hypothesis.

### Characterization of the Warburg effect in mouse lungs by confocal imaging

**Figure 4:**
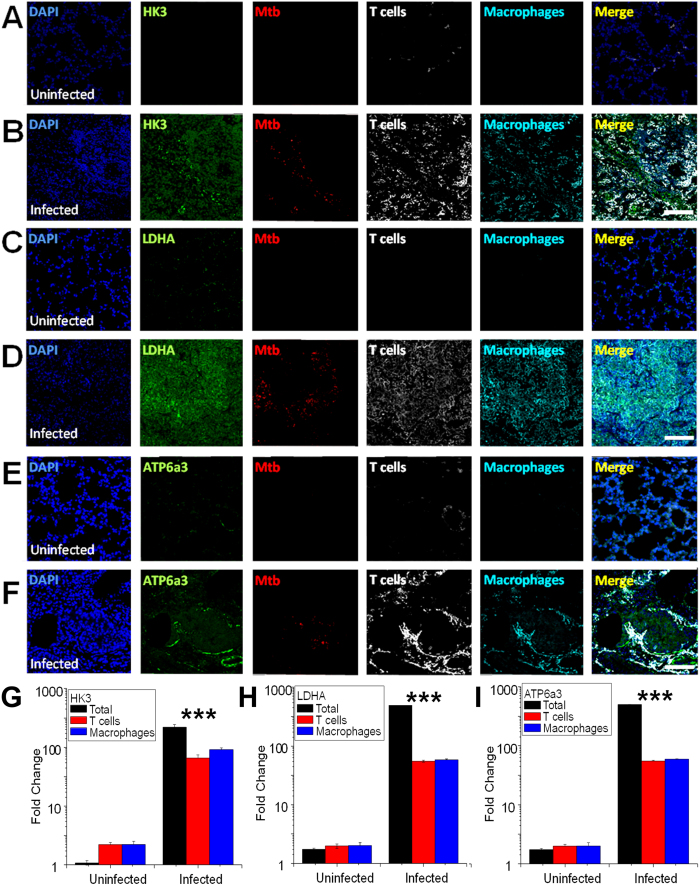
Increased expression of HK3, LDHA and ATP6a3 of V-ATPase in macrophages and CD3^+^ cells in infected mouse lungs. Confocal imaging of HK3, LDHA and ATP6a3 isoform of V-ATPase expression in macrophages and CD3^+^ cells in mouse lungs (**A–F**). Immunohistochemical staining, confocal imaging, and 3D reconstruction and analysis were carried out using 20 μm lung tissue sections obtained from uninfected control and *M. tuberculosis*-infected C57BL/6 mice at day 30 post-infection (D30). Tissue sections were stained for nuclei (DAPI, blue); HK3, LDHA, or ATP6a3 isoform of V-ATPase (green); *M. tuberculosis* (red), CD3^+^ cells (T lymphocyte, white); and IBA-1 (macrophage, cyan). Bar = 150 μm. Quantitative analysis of HK3, LDHA and ATP6a3 expression by confocal imaging in macrophages and CD3^+^ cells in lungs of infected and uninfected mice (**G–I**). The expression levels of each target protein under both conditions were obtained by measuring the positive pixels and their intensities from same number of cells of the specific regions of interests (ROIs). Three sections per lung region were examined for each animal and 4 animals per condition were analyzed. Data shown are means of fold change ± SDs of the measurements from four mice at each time point relative to background expressions from each condition. *** indicates p ≤ 0.005.

To ask whether glycolytic process is induced specifically at sites of *M. tuberculosis* infection, we used immunohistochemistry and confocal microscopy to analyze the expression of two key glycolytic enzymes, HK3 and LDHA, and of the ATP6a3 isoform of the V-ATPase in *M. tuberculosis*-containing granulomatous lesions. Thick (20 μm) sections of lung tissue from 30-day-infected and uninfected mice were used to examine granuloma-like areas in single 3D reconstructions. To associate target protein abundance with cell type in granulomas, we concurrently labeled tissue sections with antibodies against surface markers (CD3 for T cells and IBA-1 exclusively for tissue macrophages) and *M. tuberculosis* proteins^56^. The expression levels of each target protein in bacteria-containing granuloma areas were quantified by measuring the total numbers of positive pixels and intensity of each staining in all cells (total expression), or in IBA-1 or CD3 positive cells using specific region of interests (ROIs) from same number of cells under both conditions. HK3, LDHA and ATP6a3 isoform of the V-ATPase were minimally expressed in uninfected mouse lungs in all tissue sections (Fig. 4A,C,E), and increased in the granulomatous lesion area relative to uninfected control tissue (Fig. 4B,D,F). The increased signal in response to *M. tuberculosis* infection colocalized, at least in part, with macrophage and T cells (Fig. 4G–I). The enhanced expression of HK3 and LDHA in host immune cells in response to *M. tuberculosis* infection strongly suggests increased glucose uptake and glycolysis, consistent with the Warburg effect. The much higher induction of proteins compared to transcripts for these two glycolytic enzymes and ATP6a3 isoform of the V-ATPase is probably explained by the fact that protein changes are measured at the site of the infection and at the single-cell level, while mRNA measurement is derived from the whole lung tissue that includes many other cells expressing these proteins at very low levels, as in uninfected lung. The observation that the total expression of LDHA, HK3 and ATP6a3 isoform of the V-ATPase in the granulomatous lesions was higher than the sum of the expression measured in macrophages and T cells (Fig. 4G–I), is consistent with the notion that the Warburg effect also occurs in other cell types, especially those in blood vessels^57^.

### Upregulation of HIF-1α transcript and protein levels in response to *M. tuberculosis* infection

We next examined the expression of HIF-1, a transcription factor that induces genes encoding glycolytic enzymes, transporters for glucose uptake and lactate export in activated macrophages and T cells^4^,^58^. HIF-1 is a heterodimer of the highly regulated HIF-1α and the constitutively expressed HIF-1β subunits^59^. *Hif1a* activation has been associated with infection with human pathogens^60^, and with development and activation of immune cells^61^,^62^. Moreover, HIF-1-dependent glycolysis is required for Th17 cell differentiation^5^, and for production of proinflammatory mediators by macrophages^11^,^61^.

**Figure 5:**
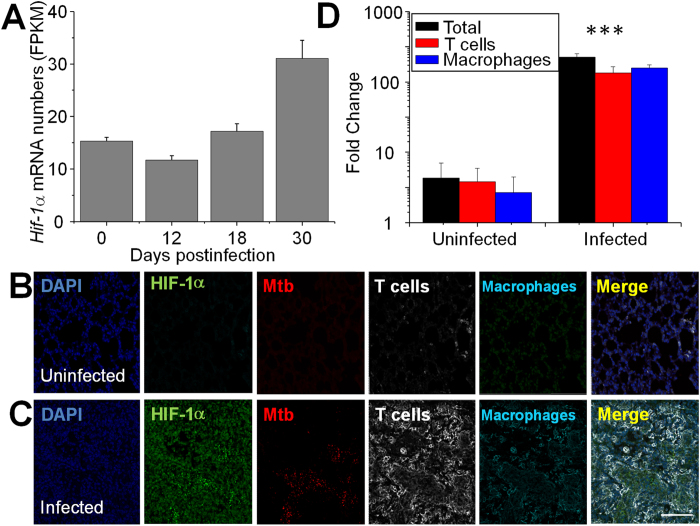
Increased *Hif1a* transcript and protein in macrophages and CD3^+^ cells in infected mouse lungs. Change of *Hif1a* transcripts during mouse lung infection (**A**). Shown are average ± SDs of the normalized *Hif1a* mRNA numbers (FPKM) from 3 mice at each time point. Changes described in text for *Hif1a* are at p ≤0.05 (student’s t-test). Confocal imaging of HIF-1α protein levels in macrophages and CD3^+^ cells in mouse lungs (**B,C**). Bar = 150 μm. Quantitative analysis of HIF-1α expression by confocal imaging in macrophages and CD3^+^ cells in lungs of infected and uninfected mice (**D)**. Immunohistochemical staining, confocal imaging, 3D reconstruction and analysis were carried out as described in Fig. 4. *** indicates p ≤ 0.005.

When we analyzed *Hif1a* transcription profiles in *M. tuberculosis*-infected mouse lungs, we found that *Hif1a* mRNA levels increased 2-fold during infection (Fig. 5A). Since HIF-1α protein is rapidly degraded under normoxic conditions^63^, mRNA induction may not lead to increased protein level. We then analyzed by confocal imaging HIF-1α protein levels in lung of uninfected mice and of mice at day 30 post-infection. While uninfected lung tissue sections showed very low levels of HIF-1α (Fig. 5B), the levels of this protein were increased in the infected tissues, and most of the increased expression colocalized with macrophages and T cells in bacilli-rich areas (Fig. 5C,D). These results strongly suggest that the Warburg effect revealed in the *M. tuberculosis*-infected mouse lung is primarily associated with these immune cells.

**Figure 6:**
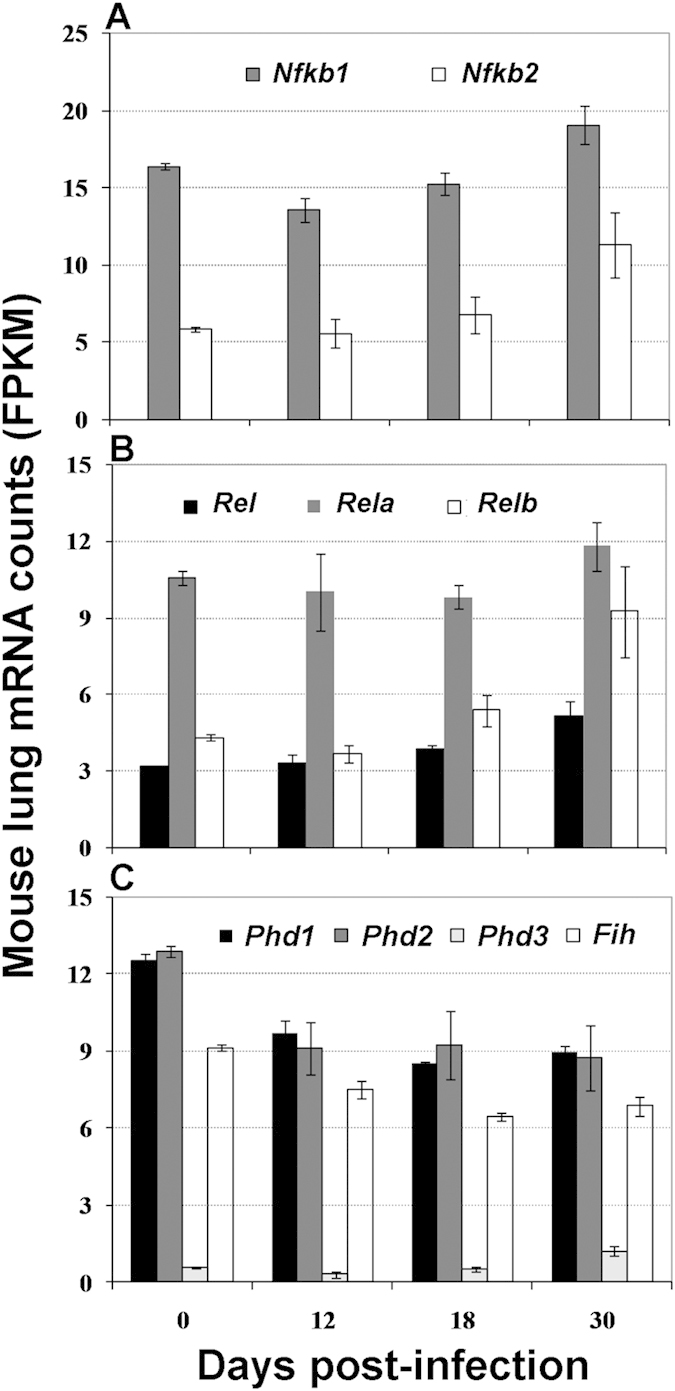
Change of transcripts encoding factors regulating *Hif1a* expression and function. Shown are average ± SDs of the normalized mRNA numbers (FPKM) from 3 mice at each time point. Each panel represents expression of genes encoding members of NF-κB family (**A,B**), and prolyl hydroxylases (PHD1-3) and aspariginyl hydroxylase known as factor inhibiting HIF (FIH) (**C**). Changes described in the text are at p ≤ 0.05 (student’s t-test) at corresponding time points.

To better understand the changes in *Hif1a* transcripts and protein, we analyzed expression of genes that mediate its regulation at the transcriptional and post-transcriptional levels. Major factors regulating *Hif1a* gene expression are members of nuclear factor-κB (NF-κB) family^64^. We found that expression of the genes encoding NF-κB1 (D30 vs D12), NF-κB2, REL, and RELB subunits increased up to 2-fold, while no significant change was observed for the gene encoding RELA (Fig. 6A,B). At the protein level, HIF-1α is regulated in multiple ways^65^,^66^. One is through inhibition of HIF-1α transactivation function by an aspariginyl hydroxylase (Factor Inhibiting HIF, or FIH)^67^. A second mechanism of HIF-α inhibition occurs through three oxygen-dependent proline hydrolases (PHD1–3), which hydroxylate HIF-α and lead to its ubiquitination and proteasomal degradation^65^. Transcripts for the highly expressed *Phd1*, *Phd2* and *Fih* were reduced by 25–30%; while *Pdh3* transcripts increased 2-fold, they were at a much lower level throughout the infection (Fig. 6C). Together, the relative changes of genes involved in *Hif1a* expression and function suggest that *M. tuberculosis* infection induces HIF-1α at the levels of expression, activity, and turnover.

## Discussion

The observed transcriptional changes of host central metabolism and the increased expression of key glycolytic isozymes and metabolic marker from confocal imaging analysis strongly suggest that transition from acute to chronic *M. tuberculosis* infection in mouse lungs, which is a consequence of Th1 immune responses^20^, is accompanied by a metabolic shift from oxidative phosphorylation toward enhanced glucose uptake, glycolysis, and formation and secretion of lactate. This bioenergetic signature is that of the Warburg effect, which is typically associated with cancer cell metabolism and regulated by HIF-1α^68^. The concurrent HIF-1α induction with host immune cells at the granulomatous lesions, together with the enhanced glycolysis and the PPP, suggests that host immune cells utilize similar bioenergetic strategy as in cancer cells for the rapid generation of ATP and biosynthetic precursors in response to *M. tuberculosis* infection^58^. Such metabolic changes are probably required to enable the synthesis of anti-microbial factors and proinflammatory mediators in response to *M. tuberculosis* infection^4^. The mechanistic link between the Warburg effect and anti-mycobacterial responses is supported by the observation that treating *M. tuberculosis*-infected THP1 cells with vitamin D3, which induces anti-mycobacterial functions^69^, results in an enhanced Warburg effect signature (transcriptomics data in GEO# GSE57028)^70^.

Given that many features of lung granulomatous lesions in C57BL/6 mouse, such as lack of hypoxia and caseous necrosis, are different from those in human tuberculosis^71^, further studies such as using models of rabbit or nonhuman primate tuberculosis that better mimic characteristics of human tuberculosis will unveil whether a similar metabolic switch also occurs in human tuberculosis. However, our observations are supported by findings from ^1^H NMR-based metabolomics profiling in lungs of *M. tuberculosis*-infected mice from a low dose aerosol model, which showed increased accumulation of lactate, the product of glycolysis^52^. Similar findings showing increased lactate accumulation in lungs of *M. tuberculosis*-infected guinea pigs with features mimicking several aspects of human tuberculosis further support our observations^53^,^54^. More importantly, the much higher induction of glycolytic enzymes and related metabolic marker from the confocal imaging analysis in macrophages and T cells of the granulomatous lesions than that of the transcriptomics profiling from the whole lung further supports the notion that the Warburg effect occurs mainly in host immune cells of granulomatous lesions. Particularly, the induction of HKs, especially HK3 that plays key regulatory roles in glucose metabolism and is essential to glucose uptake by maintaining the glucose gradient for glucose uptake mediated by the facilitative GLUTs^26^, underscores the enhanced glucose metabolism during *M. tuberculosis* infection. Thus, our data are consistent with the findings from 2-deoxy-2-[^18^F]-fluoro-D-glucose (FDG) positron emission tomography/computed tomography (PET/CT) imaging that metabolic state of host immune cells, as measured by FDG uptake in human tuberculosis patients and infected nonhuman primates, is associated with disease states^72^. The concurrent upregulation of the PPP and the Warburg effect is also in agreement with the metabolic state of activated immune cells^1^,^44^. It is also interesting to understand whether the sedoheptulose kinase (CARKL), an orphan enzyme in the PPP that regulates glycolysis and glucose metabolism during macrophage polarization^44^, also plays similar roles during *M. tuberculosis* infection (no mRNA profiles for CARKL were identified in mouse lungs).

The observed expression patterns and isozyme preferences for glucose transporters, glycolytic enzymes, and lactate/pyruvate transporters in the tuberculous mouse lung likely result in a coordinated process occurring under specific metabolic conditions and presumably in specific cell types. For example, among the hexokinases, we observed strong upregulation only of HK3, the isoform expressed in few tissues including lung^26^,^73^,^74^. Since HK3 is the isoform with the highest affinity for glucose and the lowest sensitivity to inhibition by the end product G-6-P^26^, its upregulation might satisfy a metabolic requirement for maximal conversion of glucose to G-6-P during *M. tuberculosis* infection. This idea is supported by the observation that *Hk3* and *Glut6* exhibit similar expression patterns, suggesting that these two enzymes function together to maximize glucose import. Another example is provided by the MCTs, which likely regulate the relative efflux of their substrates, lactate and pyruvate, on the basis of substrate affinity patterns^45^, and the relative expression levels of each isoform. In *M. tuberculosis*-infected lungs, downregulation of MCT2, which has the lowest Km with pyruvate and lactate, may prevent depletion of pyruvate, while upregulation of MCT4, which has the highest Km for pyruvate and a much lower Km for lactate, may limit pyruvate efflux while maintaining a high rate of lactate efflux^75^. As a result, these MCTs would facilitate the Warburg effect by maintaining the optimal concentration of pyruvate (the substrate of lactate dehydrogenase), while exporting lactate to keep cytosolic pH around physiological levels.

Our data also point to a temporal regulation of the Warburg effect involving HIF-1α. Increased levels of protein likely reflect both increased gene expression and stabilization through reduced expression of PHDs that hydroxylate HIF-1α and drive its turnover. Stabilization of HIF-1α could also be enhanced by reduced oxygen supply in the granulomatous microenvironment due to inflammation^76^, and by the accumulation of TCA cycle intermediates^77^, such as succinate^78^, both of which would further inactivate the PHDs. Succinate was indeed increased in *M. tuberculosis*-infected mouse lungs^52^. In addition, since iron serves as a cofactor for the hydroxylation reaction by the PHDs, iron deficiency due to sequestration by *M. tuberculosis* siderophores^79^, could contribute to the inhibition of these hydrolases leading to the HIF-1α accumulation in mouse lungs. Moreover, we observed that the two homologous transcription factors HIF-1α and HIF-2α, which have physiologically antagonistic functions^80^, have divergent expression patterns (Supplemental Fig. S4), consistent with the importance of increased HIF-1α expression and function in the induction of the Warburg effect.

The temporal association of HIF-1α expression and the Warburg effect in host immune cells during *M. tuberculosis* infection warrants further in-depth study, considering the complexity of host immune cells with different types/subtypes in granulomas and the dynamic effects of evolving granuloma-associated microenvironments on the relative representation and activation state of these immune cells. The contribution of other signaling pathways including negative AMPK regulator to the cellular metabolism^4^, should also be taken into account. In addition, since extracellular acidification has profound effect on cellular metabolism and functions^81^,^82^, it is worth investigating the consequence of the Warburg effect for the metabolism and functions of different immune cells in granulomas. With the consideration that extracellular lactate can be uptaken and reutilized as carbon and energy source^83^, it is also interesting to study the metabolic fate of extracellular lactate in granulomas to understand whether different immune cells including infected macrophages have different ability to produce, transport and metabolize lactate. Understanding the metabolic shifts of granuloma-associated immune cells and the regulatory underpinnings will contribute to our ability to manipulate the energy metabolism of innate and adaptive immune cells as a new anti-tuberculosis immunotherapy.

## Materials and Methods

### Mice

C57BL/6 mice were purchased from The Jackson Laboratory (Bar Harbor, Maine). Female mice at 8 weeks of age were used for experiments. All procedures involving live animals were performed in accordance with the Guide for Care and Use of Laboratory Animals of the National Institutes of Health, and individual procedures were approved by the Trudeau Institute Institutional Animal Care and Use Committee.

### Bacterial culture and aerosol infection

*M. tuberculosis* strain H_37_Rv (Trudeau Mycobacterial Culture Collection no. 102) was grown in Proskauer Beck medium containing 0.05% Tween 80 to mid-log phase and frozen in 1 ml of aliquots at -80 °C. Mice were infected with ~75 CFU of bacteria using a Glas-Col airborne infection system as described^19^. Three mice were sacrificed on day 1 of infection to verify the inoculum in the mouse lungs. Lungs from three uninfected control mice and three mice from days 12, 18, and 30 post-infection were harvested, snap-frozen in liquid nitrogen and kept at −80 °C until use.

### Total RNA extraction and depletion of rRNAs

Total RNA extraction was carried out as described^20^. rRNA depletion from total RNA preparations was performed using the Ribozero kits for mouse and gram-positive bacteria (Epicentre, Madison, WI) according to manufacturer’s recommendations. To establish that rRNA depletion did not skew measurements, we used qRT-PCR to quantify *Ifng* transcripts before and after rRNA depletion and found that changes of normalized *Ifng* transcripts in infected mouse lungs relative to uninfected controls in rRNA-depleted samples were similar to those in non-depleted samples (Supplemental Fig. S1). Procedures and conditions for reverse transcription and real-time PCR quantification of host gene expression with molecular beacons were carried out as described^20^. Sequences of PCR primers and molecular beacons are for *Ifng*: forward: 5′-GGCCATCAGCAACAACATAAGC-3′; reverse: 5′-TGACCTCAAACTTGGCAATACTCA-3′; and molecular beacon: 5′-fluorophore-AGCGCGCTACCTTCTTCAGCAACAGCAAGGCGACGCGCT-quencher-3′; and for *Gapdh*: forward: 5′-CTCTGGAAAGCTGTGGCGTGATG-3′; reverse: 5′-GTTTCTCCAGGCGGCACGTC-3′; and molecular beacon: 5′-fluorophore-ACCGCCAAGGTCATCCCAGAGCTGAACGGGGCGGT-quencher-3′.

### RNA sequencing

RNA sequencing (RNA-Seq) was carried out by BGI Americas (Cambridge, MA) using Illumina platform with TruSeq v2 with 150 – 200 bp short insert library construction and 91 bp pair-end sequencing. Briefly, rRNA-depleted mRNA samples were subjected to fragmentation followed by reverse transcription for the first strand cDNA synthesis; after the second strand cDNA synthesis, the double strand (ds) cDNAs were repaired and adenylated for the ligation of multiple indexing adapters with AT complementary overhang to the ends of ds-cDNAs; after enrichment of the ds-DNA library by 15 cycles of PCR, the library was validated and sequenced with 91 bp pair-end reads. About 8GB of data (corresponding to about 40 million reads) were obtained per sample.

### Bioinformatics analysis

Using Tophat v2.0.6 (http://tophat.cbcb.umd.edu/) and *Mus musculus* NCBI build37.2 from iGenomes (http://cufflinks.cbcb.umd.edu/igenomes.html), more than 90% of mapped reads properly paired to the mouse genome for each sample. Expression was estimated in each sample by Fragments Per Kilobase of exon per Million fragments (FPKM) for all RefSeq transcripts (as supplied by iGenomes genes.gtf annotations) using cufflinks 2.0.2 (http://cufflinks.cbcb.umd.edu/) on a Linux Debian installation.

### Statistical analysis

Student’s T-tests, Benjamin-Hochberg false discovery rate corrections, and ratio estimates were calculated in R (http://www.R-project.org) for differential expression as measured by FPKM values. Expression ratio estimates were calculated as the antilog of difference between mean log values. Expression changes with p value ≤ 0.05 by the Student’s T-tests were considered to be significant.

### Immunofluorescence microscopy

Lungs from uninfected control mice and *M. tuberculosis*-infected mice from day 30 post-infection were fixed in 10% neutral buffered formalin (Sigma), embedded into paraffin blocks and cut into thick sections (20 μm). Lung tissue sections were analyzed by five-color immunohistochemical staining with antibodies for nuclei: DAPI; *M. tuberculosis*: biotin antibody: Genetex, Irvine, CA; hexokinase 3 (HK3): Biorbyt, Cambridge, UK; subunit isoform a3 of V0 domain of V-type H^+^ ATPase (ATP6a3): Santa Cruz, Dallas, TX; lactate dehydrogenase A (LDHA), or HIF-1α: Abcam, Cambridge, MA; macrophage: ionized calcium-binding adapter molecule 1 (IBA-1), a marker exclusively for tissue macrophages, Abcam; and CD3: a marker for lymphocytes, Abcam. Analysis was performed using an A1 confocal microscope equipped with spectrum detection and unmixing properties as we recently described^56^. Antibody specificity and autofluorescence were confirmed by replacing the primary antibody with a non-specific myeloma protein of the same isotype or non-immune serum as well as calculating the intensity of a pan-antibody, IBA-1, as we previously described^56^,^84^. The expression level and distribution of target proteins were analyzed using the 3D reconstructions and deconvolution followed by the generation of regions of interests (ROIs). Using the corresponding ROIs the quantitative expression analysis of each target protein was determined by quantifying the intensity of positive pixels for the whole deconvoluted 3D reconstruction or for the positive pixels in IBA-1 positive cells and CD3^+^ cells. For the differential analysis of each target protein between infected and uninfected controls, same number of cells (300 to 800 cells) from specific ROIs in both conditions was analyzed to avoid bias due to increased cell density in infected lung tissues. Three lung sections from each animal and four animals at each time point were analyzed. Mean differences were tested by non-parametric Kruscal-Wallis analysis. If a significant F-value was obtained, means were compared with Bofferonni-Dunn multiple comparison test. A value of p ≤ 0.05 was considered significant. Details of the procedure, including deparaffinization, antigen retrieval, blocking, immunohistochemical staining with primary and secondary antibodies, and specificity testing, were previously described^56^.

## Additional Information

**How to cite this article**: Shi, L. *et al.* Infection with *Mycobacterium tuberculosis *induces the Warburg effect in mouse lungs. *Sci. Rep.* **5**, 18176; doi: 10.1038/srep18176 (2015).

## Supplementary Material

Supplementary Information
