# Supplementary material for: Infection with Mycobacterium tuberculosis induces the Warburg effect in mouse lungs
Source: Sci Rep. 2015 Dec 10;5:18176. doi: 10.1038/srep18176 (PMC4674750; doi:10.1038/srep18176)
Supplement: Supplementary Information [file srep18176-s1.pdf]

Supplemental files:

Infection with *Mycobacterium tuberculosis* induces the Warburg effect in mouse lungs

Lanbo Shi[1,*], Hugh Salamon[2], Eliseo A. Eugenin[1], Richard Pine[1], Andrea Cooper[3] and Maria L. Gennaro[1]

[1]Public Health Research Institute, New Jersey Medical School, Rutgers, The State University of New Jersey, Newark, NJ, USA

[2]Knowledge Synthesis Inc. Berkeley, CA, USA

[3]Trudeau Institute, Saranac Lake, NY, USA

*Corresponding author: shila@njms.rutgers.edu

Running title:

The Warburg effect in *M. tuberculosis*-infected mouse lungs

Supplemental Figure S1

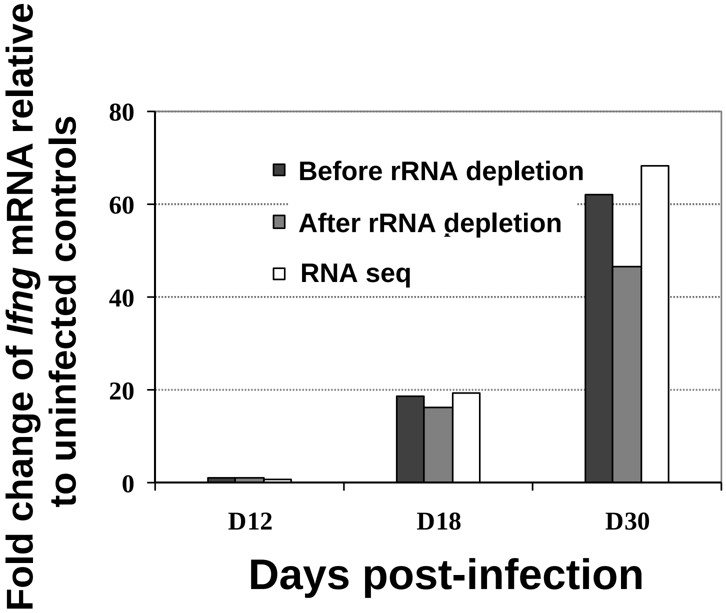

Supplemental Fig. S1. Change of *Ifng* transcripts in infected mouse lungs. *Ifng* transcripts were measured by RT-PCR using molecular beacons before and after rRNA depletion and by RNA-seq. Shown are average fold change of normalized *Ifng* transcripts from lungs of 3 mice at each time points relative to uninfected control mice. RT-PCR data were normalized with the expression level of *Gapdh* in the same samples. RNA-seq data were derived from normalized mRNA counts (FPKM) from 3 mice at each time point.

Supplemental Figure S2

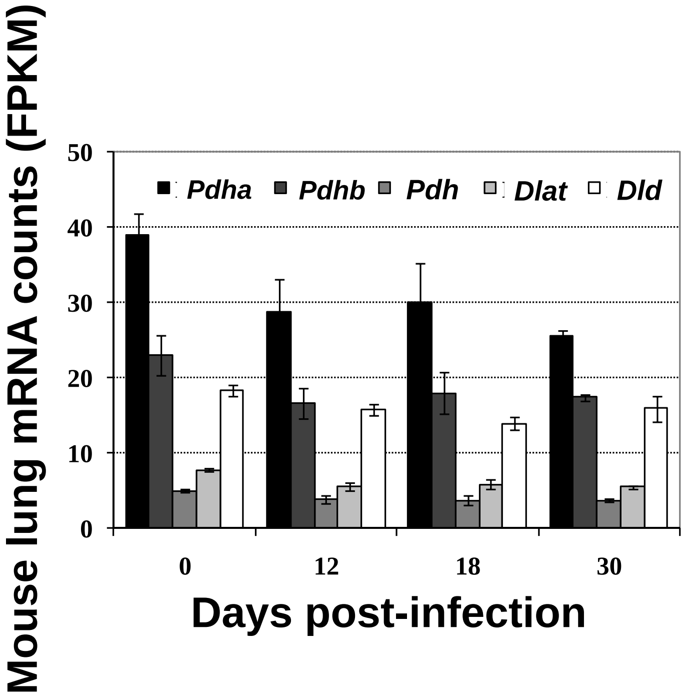

Supplemental Fig. S2. Change of transcripts encoding pyruvate dehydrogenase complex subunits in infected mouse lungs. Shown are average ± SDs of the normalized mRNA counts (FPKM, Fragments Per Kilobase of exon per Million fragments mapped) from 3 mice at each time point. Changes described in the text are at $p \leq 0.05$ (student's t-test) at corresponding time points.

Supplementary Figure S3

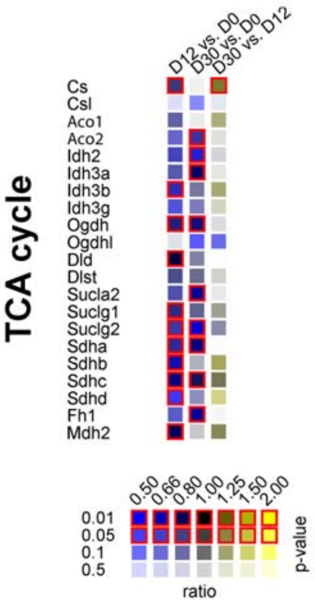

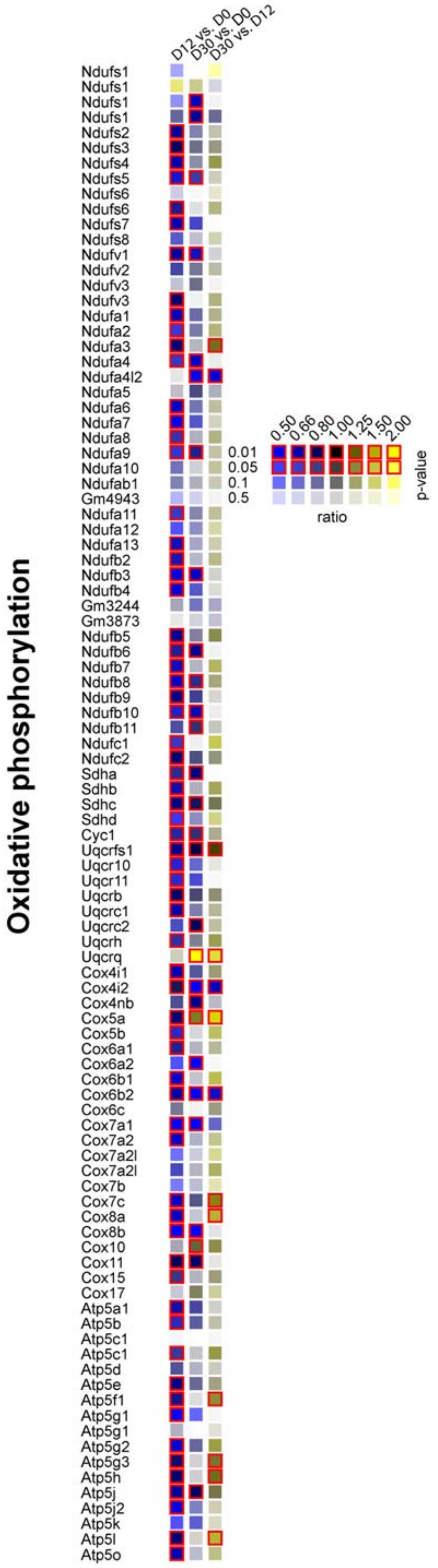

Supplemental Fig. S3. Heatmap analysis of transcripts encoding enzymes/components mediating TCA cycle and oxidative phosphorylation in infected mouse lungs. Normalized expression (FPKM) for all RefSeq transcripts (as supplied by iGenomes genes.gtf annotations) from three mice at each time point were obtained as described in Materials and Methods. Expression ratio estimates at different time points calculated as the antilog of difference between mean log values are shown. The legend indicates changes in expression (different colors, across), and significance (different shades, down). Expression changes with p value ≤ 0.05 by the Student's T-tests are shown with red borders. Multiple rows labeled for a single gene represent data derived from different mRNA splice variants of that gene.

Supplementary Figure S4

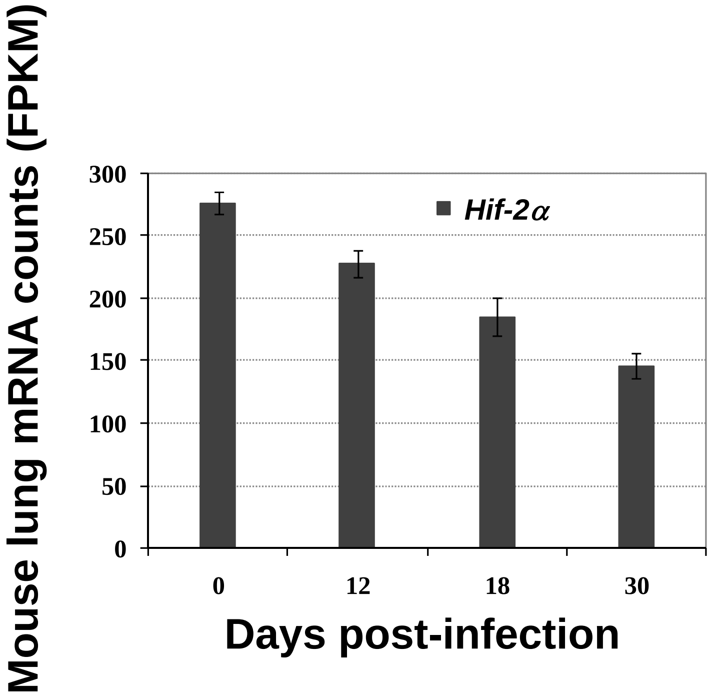

Supplementary Fig. S4. Change of *Hif-2α* transcripts in infected mouse lungs. Shown are average ± SDs of the normalized mRNA counts (FPKM, Fragments Per Kilobase of exon per Million fragments mapped) from 3 mice at each time point. Changes described in the text are at $p \leq 0.05$ (student's t-test) at corresponding time points.

Supplemental Table S1. Changes of transcripts encoding facilitative glucose transporters, glycolytic enzymes, monocarboxylic acid transporters (MCTs), subunits of V-ATPase and pentose phosphate pathway in mouse lungs.

Entrez	Symbol	Transcript_id	Ratio (D12/D0)	P value	Ratio (D30/D12)	P value	Ratio (D30/D0)	P value
**Facilitative glucolse transporters**								
20525	*Slc2a1 (Glut1)*	NM_011400.3	0.871	0.202	1.803	0.032	1.571	0.077
20526	*Slc2a2 (Glut2)*	NM_031197.2	0.497	0.153	0.893	0.777	0.444	0.047
20527	*Slc2a3 (Glut3)*	NM_011401.4	0.623	0.011	1.977	0.004	1.232	0.125
20528	*Slc2a4 (Glut4)*	NM_009204.2	0.492	0.019	0.667	0.083	0.328	0.001
56485	*Slc2a5 (Glut5)*	NM_019741.3	0.827	0.809	0.667	0.647	0.552	0.359
227659	*Slc2a6 (Glut6)*	NM_001177627.1	0.000	0.102	1653.710	0.192	0.535	0.906
227659	*Slc2a6 (Glut6)*	NM_172659.2	1.090	0.437	6.317	0.005	6.884	0.003
56017	*Slc2a8 (Glut8)*	NM_019488.4	0.901	0.472	0.837	0.268	0.754	0.026
**Glycolysis**								
103988	*Gck*	NM_010292.4	0.703	0.394	1.134	0.745	0.798	0.190
72141	*Adpgk*	NM_028121.2	0.909	0.280	1.080	0.416	0.981	0.781
216019	*Hkdc1*	NM_145419.1	0.757	0.168	1.116	0.582	0.844	0.299
15275	*Hk1*	NM_001146100.1	0.767	0.003	1.403	0.026	1.076	0.391
15275	*Hk1*	NM_010438.3	0.990	0.977	0.942	0.651	0.933	0.847
15277	*Hk2*	NM_013820.3	0.729	0.011	2.191	0.007	1.597	0.039
212032	*Hk3*	NM_001033245.3	0.777	0.168	20.890	0.001	16.232	0.002
66681	*Pgm1*	NM_025700.2	0.851	0.084	1.192	0.169	1.014	0.889
72157	*Pgm2*	NM_028132.3	0.711	0.068	1.464	0.057	1.040	0.735
14751	*Gpi1*	NM_008155.3	0.793	0.084	1.509	0.033	1.197	0.210
18641	*Pfkl*	NM_008826.4	0.716	0.051	1.509	0.029	1.080	0.405
18642	*Pfkm*	NM_021514.4	0.015	0.423	17590.100	0.127	271.267	0.183
18642	*Pfkm*	NM_001163487.1	0.770	0.075	0.673	0.016	0.518	0.004
18642	*Pfkm*	NM_001163488.1	0.314	0.786	5.766	0.620	1.810	0.886
56421	*Pfkp*	NM_019703.3	0.781	0.035	2.011	0.006	1.571	0.035
14121	*Fbp1*	NM_019395.2	0.948	0.832	0.391	0.023	0.371	0.024
14120	*Fbp2*	NM_007994.3	0.494	0.060	1.805	0.196	0.891	0.780
18639	*Pfkfb1*	NM_008824.2	0.896	0.766	1.425	0.380	1.276	0.083
18640	*Pfkfb2*	NR_027859.1	0.971	0.936	1.124	0.759	1.091	0.747
18640	*Pfkfb2*	NM_008825.4	0.899	0.009	1.057	0.170	0.950	0.194
18640	*Pfkfb2*	NM_001162415.1	0.888	0.297	0.933	0.524	0.828	0.205
170768	*Pfkfb3*	NM_001177752.1	1.112	0.707	0.769	0.410	0.854	0.577
170768	*Pfkfb3*	NM_001177755.1	0.685	0.523	3.274	0.046	2.244	0.202
170768	*Pfkfb3*	NM_001177758.1	1.481	0.492	1.328	0.615	1.967	0.221
170768	*Pfkfb3*	NM_133232.3	0.245	0.237	5.321	0.184	1.305	0.178
270198	*Pfkfb4*	NM_173019.5	0.833	0.111	1.171	0.233	0.975	0.796
11674	*Aldoa*	NM_001177307.1	0.777	0.072	1.410	0.049	1.095	0.438
11674	*Aldoa*	NM_001177308.1	0.975	0.908	0.786	0.325	0.767	0.064
11674	*Aldoa*	NM_007438.4	0.643	0.130	1.627	0.120	1.047	0.809

Supplemental Table S1 continued:

Entrez	Symbol	Transcript_id	Ratio (D12/D0)	P value	Ratio (D30/D12)	P value	Ratio (D30/D0)	P value
**Glycolysis**								
230163	*Aldob*	NM_144903.2	0.764	0.617	1.466	0.690	1.121	0.901
11676	*Aldoc*	NM_009657.3	0.710	0.138	2.170	0.016	1.542	0.058
21991	*Tpi1*	NM_009415.2	0.656	0.111	1.978	0.035	1.298	0.195
14433	*Gapdh*	NM_008084.2	0.777	0.042	2.195	0.022	1.705	0.053
18655	*Pgk1*	NM_008828.2	0.714	0.008	2.146	0.006	1.533	0.037
12183	*Bpgm*	NM_007563.4	0.603	0.045	1.027	0.863	0.620	0.015
18648	*Pgam1*	NM_023418.2	0.749	0.031	1.847	0.022	1.383	0.069
56012	*Pgam2*	NM_018870.3	0.489	0.021	0.933	0.683	0.457	0.017
13806	*Eno1*	NM_023119.2	0.791	0.126	2.175	0.003	1.720	0.011
13807	*Eno2*	NM_013509.2	0.677	0.063	1.268	0.180	0.859	0.261
13808	*Eno3*	NM_007933.2	0.778	0.119	1.080	0.628	0.841	0.245
13808	*Eno3*	NM_001136062.1	0.472	0.019	1.035	0.854	0.489	0.004
226265	*Eno4*	NM_178689.4	0.906	0.178	0.744	0.004	0.674	0.004
433182	*Gm5506*	NM_001025388.1	0.744	0.102	2.212	0.000	1.645	0.030
18770	*Pklr*	NM_001099779.1	1.750	0.257	0.794	0.700	1.390	0.609
18746	*Pkm*	NM_011099.2	0.719	0.030	1.863	0.025	1.340	0.152
16828	*Ldha*	NM_001136069.2	1.058	0.851	1.592	0.196	1.684	0.219
16828	*Ldha*	NM_010699.2	0.836	0.054	1.717	0.011	1.434	0.022
106557	*Ldhal6b*	NM_175349.2	1.191	0.095	1.265	0.286	1.507	0.116
16832	*Ldhb*	NM_008492.2	0.686	0.016	0.830	0.100	0.569	0.003
16833	*Ldhc*	NM_013580.4	1.390	0.669	NA	NA	NA	NA
**Pentose phosphate pathway**								
14751	*Gpi1*	NM_008155.3	0.793	0.084	1.509	0.033	1.197	0.210
14381	*G6pdx*	NM_008062.2	0.822	0.035	1.497	0.036	1.231	0.150
100198	*H6pd*	NM_173371.3	0.761	0.040	0.912	0.319	0.694	0.008
66171	*Pgls*	NM_025396.3	0.701	0.097	1.394	0.159	0.977	0.890
110208	*Pgd*	NM_001081274.1	0.998	0.980	1.397	0.048	1.394	0.053
21881	*Tkt*	NM_009388.5	0.783	0.187	1.144	0.501	0.896	0.563
83553	*Tktl1*	NM_031379.2	0.870	0.583	1.093	0.677	0.951	0.805
21351	*Taldo1*	NM_011528.4	0.659	0.009	1.444	0.039	0.952	0.683
66646	*Rpe*	NM_025683.2	0.880	0.129	1.114	0.179	0.980	0.644
19895	*Rpia*	NM_009075.2	0.702	0.021	1.247	0.069	0.875	0.072
232449	*Dera*	NM_172733.1	0.911	0.467	1.561	0.023	1.422	0.026
**Monocarboxylic acid transporters**								
20501	*Slc16a1 (Mct1)*	NM_009196.3	0.912	0.149	1.041	0.388	0.949	0.331
20503	*Slc16a7 (Mct2)*	NM_011391.1	0.662	0.015	0.849	0.195	0.562	0.006
57274	*Slc16a8 (Mct3)*	NM_020516.2	0.563	0.402	0.853	0.806	0.480	0.103
80879	*Slc16a3 (Mct4)*	NM_030696.3	0.075	0.525	40.681	0.388	3.063	0.017
80879	*Slc16a3 (Mct4)*	NM_001038653.1	0.059	0.396	1.276	0.012	2.685	0.048
80879	*Slc16a3 (Mct4)*	NM_001038654.1	0.014	0.392	9.323	0.717	0.129	0.720

Supplemental Table S1 continued:

Entrez	Symbol	Transcript_id	Ratio (D12/D0)	P value	Ratio (D30/D12)	P value	Ratio (D30/D0)	P value
**V-H$^+$ ATPase**								
11964	*Atp6v1a*	NM_007508.5	0.755	0.011	1.162	0.064	0.877	0.102
110935	*Atp6v1b1*	NM_134157.2	0.561	0.046	0.580	0.096	0.325	0.026
11966	*Atp6v1b2*	NM_007509.3	0.905	0.024	1.285	0.002	1.163	0.007
66335	*Atp6v1c1*	NM_025494.3	0.797	0.085	1.287	0.056	1.025	0.587
68775	*Atp6v1c2*	NM_001159632.1	0.880	0.198	0.677	0.004	0.595	0.005
68775	*Atp6v1c2*	NM_133699.2	0.630	0.200	0.757	0.385	0.477	0.014
73834	*Atp6v1d*	NM_023721.2	0.793	0.013	1.431	0.004	1.135	0.104
11973	*Atp6v1e1*	NM_007510.2	0.740	0.037	1.255	0.075	0.929	0.391
66144	*Atp6v1f*	NM_025381.2	0.570	0.027	1.521	0.093	0.866	0.435
66290	*Atp6v1g1*	NM_024173.2	0.655	0.028	1.164	0.282	0.763	0.100
66237	*Atp6v1g2*	NM_023179.3	2.380	0.165	0.639	0.111	1.519	0.426
338375	*Atp6v1g3*	NM_177397.3	0.830	0.813	0.645	0.416	0.535	0.479
108664	*Atp6v1h*	NM_133826.4	0.703	0.024	1.242	0.044	0.873	0.081
11975	*Atp6v0a1*	NM_016920.2	0.945	0.049	0.869	0.003	0.822	0.001
21871	*Atp6v0a2*	NM_011596.4-1	0.852	0.016	1.464	0.023	1.247	0.086
21871	*Atp6v0a2*	NM_011596.4-2	0.876	0.139	1.419	0.054	1.244	0.156
27060	*Tcirg1 (Atp6a3)*	NM_001167784.1	1.416	0.335	1.728	0.153	2.448	0.075
27060	*Tcirg1 (Atp6a3)*	NM_001136091.2	1.071	0.665	2.672	0.007	2.862	0.004
27060	*Tcirg1 (Atp6a3)*	NM_016921.3	20.087	0.526	3.361	0.008	67.501	0.397
140494	*Atp6v0a4*	NM_080467.3	0.937	0.427	0.572	0.005	0.536	0.000
114143	*Atp6v0b*	NM_033617.3	0.799	0.007	1.255	0.077	1.003	0.972
11984	*Atp6v0c*	NM_009729.3	1.083	0.503	1.696	0.020	1.837	0.001
11972	*Atp6v0d1*	NM_013477.3	0.757	0.007	1.268	0.036	0.960	0.599
242341	*Atp6v0d2*	NM_175406.3	1.005	0.979	1.428	0.182	1.435	0.036
11974	*Atp6v0e*	NM_025272.2	0.739	0.011	1.453	0.063	1.075	0.575
76252	*Atp6v0e2*	NM_133764.3	0.809	0.017	0.815	0.000	0.660	0.005

Normalized expression (FPKM) was determined as described in the Materials and Methods for all RefSeq transcripts (as supplied by iGenomes genes.gtf annotations) from three mice at each time point. Shown are expression ratio estimates at different time points calculated as the antilog of difference between mean log values. Very high ratios marked with grey background were artifacts of very low expression of the related transcripts in mouse lungs at the corresponding control time point, and are thus deemed to lack biological meaning. NA indicates values could not be determined.
